# Supplementary material for: Dairy Cows’ Udder Pathogens and Occurrence of Virulence Factors in Staphylococci
Source: Animals (Basel). 2022 Feb 14;12(4):470. doi: 10.3390/ani12040470 (PMC8868196; doi:10.3390/ani12040470)
Supplement: Supplementary file 1 [file animals-12-00470-s001.zip › animals-1468204-supplementary.pdf]

Supplementary materials

Table S1. An overview of the most commonly used intramammary antimicrobials for dry cow therapy.

**Product.**	**Active ingredients**	**Category**
Kloxerate Plus DC	Cloxacillinum 500 mg<br>Ampicillinum 250 mg	DC
Bovaclox DC Extra	Cloxacillinum 600 mg<br>Ampicillinum 300 mg	DC
Noroclox DC Extra	Benzathine cloxacillinum 600 mg	DC
Nafpenzal DC	Benzylpenicillinum 300 000 IU<br>Nafcillinum 100 mg<br>Dihydrostreptomycinum100 mg	DC
Cefquinor DC IMM	Cefquinome 150 mg	DC
Cepravin DC	Cefalonium 250 mg	DC
Orbenin DC	Cloxacillin Benzathine 500 mg	DC
Orbenin Extra DC	Benzathine Cloxacillin (equivalent to cloxacillin) 600 mg	DC
Ubrostar Red DC	Benethamine Penicillin 280 mg, Framycetin Sulfate 100 mg, Penethamate Hydriodide 100 mg	DC
Spectramast DC	Ceftiofur Equivalents (as the hydrochloride salt) 500 mg	DC
Quadran DC	Cephalonium 250 mg	DC
ToMorrow	Cephapirin 300 mg	DC
Quartermaster® Suspension	Procaine Penicillin G 10^6 IU Dihydrostreptomycin Sulfate 1000 mg	DC

Note: DC – dry cows.
